# Portal vein thrombosis after cesarean section in a patient on prolonged bed rest due to threatened preterm labor

**DOI:** 10.1002/ccr3.1405

**Published:** 2018-02-06

**Authors:** Arata Hibi, Kazumasa Mogi

**Affiliations:** ^1^ Division of Nephrology and Rheumatology Department of Internal Medicine Kariya Toyota General Hospital 5‐15, Sumiyoshi‐cho Kariya Aichi 448‐8505 Japan; ^2^ Department of Obstetrics and Gynecology Kariya Toyota General Hospital 5‐15, Sumiyoshi‐cho Kariya Aichi 448‐8505 Japan

**Keywords:** Cesarean section, portal venous thrombosis, pregnancy

## Abstract

Portal vein thrombosis is a rare but life‐threatening complication during pregnancy and postpartum period. Color Doppler ultrasound is useful for prompt diagnosis. Although the risk of complications should be considered, successful pregnancy with comorbid portal vein thrombosis is possible with appropriate anticoagulation therapy and close monitoring.

## Introduction

Portal vein thrombosis (PVT) refers to complete or partial obstruction of the portal venous system due to thrombus formation 1. Cirrhosis and hypercoagulable conditions, such as malignancies, infections, and hematologic disorders, account for most cases of PVT 2. Women of childbearing age account for approximately 25% of cases with noncirrhotic PVT 3. Although pregnancy is a condition with hypercoagulability and portal hypertension 4, 5, PVT is a rare complication during pregnancy and postpartum period 6. However, cesarean section (CS) is a common predisposing factor for deep venous thrombosis (DVT) 7 while prolonged bed rest is not significantly associated with the risk of DVT 8. In patients with acute PVT, immediate anticoagulation therapy is critical for recanalization because intestinal perforation due to ischemia and infarction is a serious complication of PVT 9. The diagnosis of acute PVT is challenging because it may manifest as acute abdomen. Contrary to acute PVT, chronic PVT is usually asymptomatic and sometimes incidentally found on an ultrasound examination during pregnancy 10. However, in patients with chronic PVT, variceal bleeding from the upper gastrointestinal (GI) tract due to consistent portal hypertension is one of the most serious complications during pregnancy 5. Importantly, there are no definite guidelines at present for the management of acute or chronic PVT during pregnancy. We encountered an interesting case of a primigravida patient with acute PVT after CS. Although complete recanalization was not obtained, she achieved a second successful delivery with continuous anticoagulation therapy during pregnancy. We propose the importance of early detection and immediate anticoagulation therapy for acute PVT and necessity of close monitoring in pregnant women with chronic PVT which is controlled with anticoagulation therapy.

## Case Report

**Figure 1:**
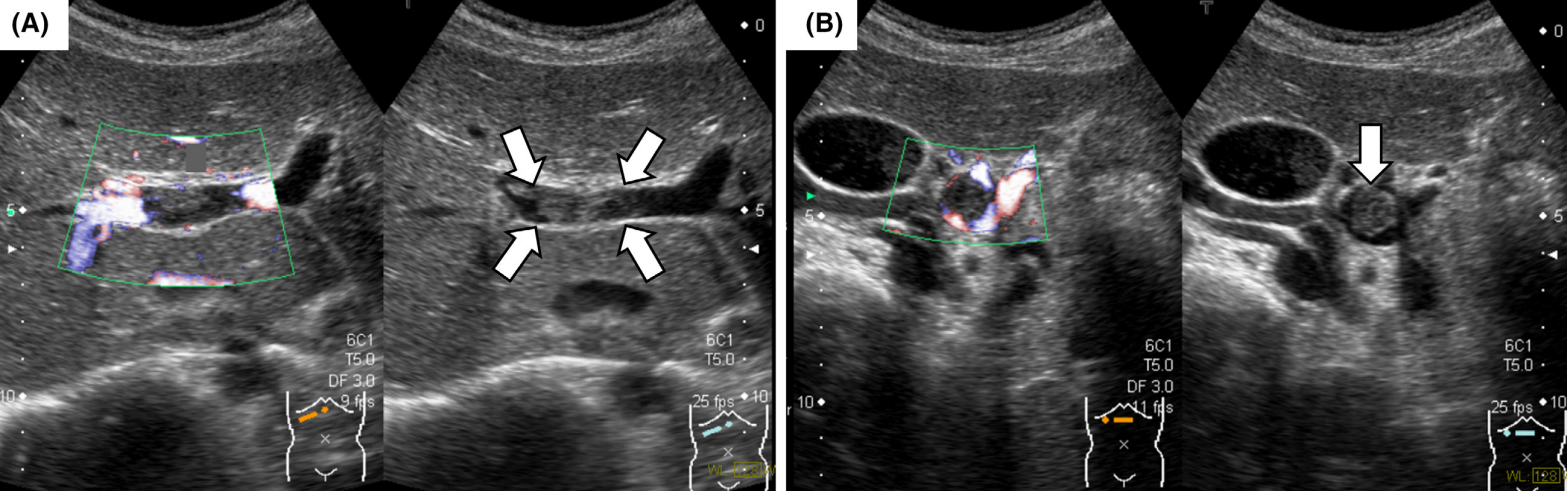
Abdominal ultrasonography results on the day of admission. (A) A massive intrahepatic thrombus is seen, and blood flow is barely detected on color Doppler sonography (left). (B) The superior mesenteric vein is dilated and occluded by a massive thrombus. No blood flow is detected on color Doppler sonography (left).

**Figure 2:**
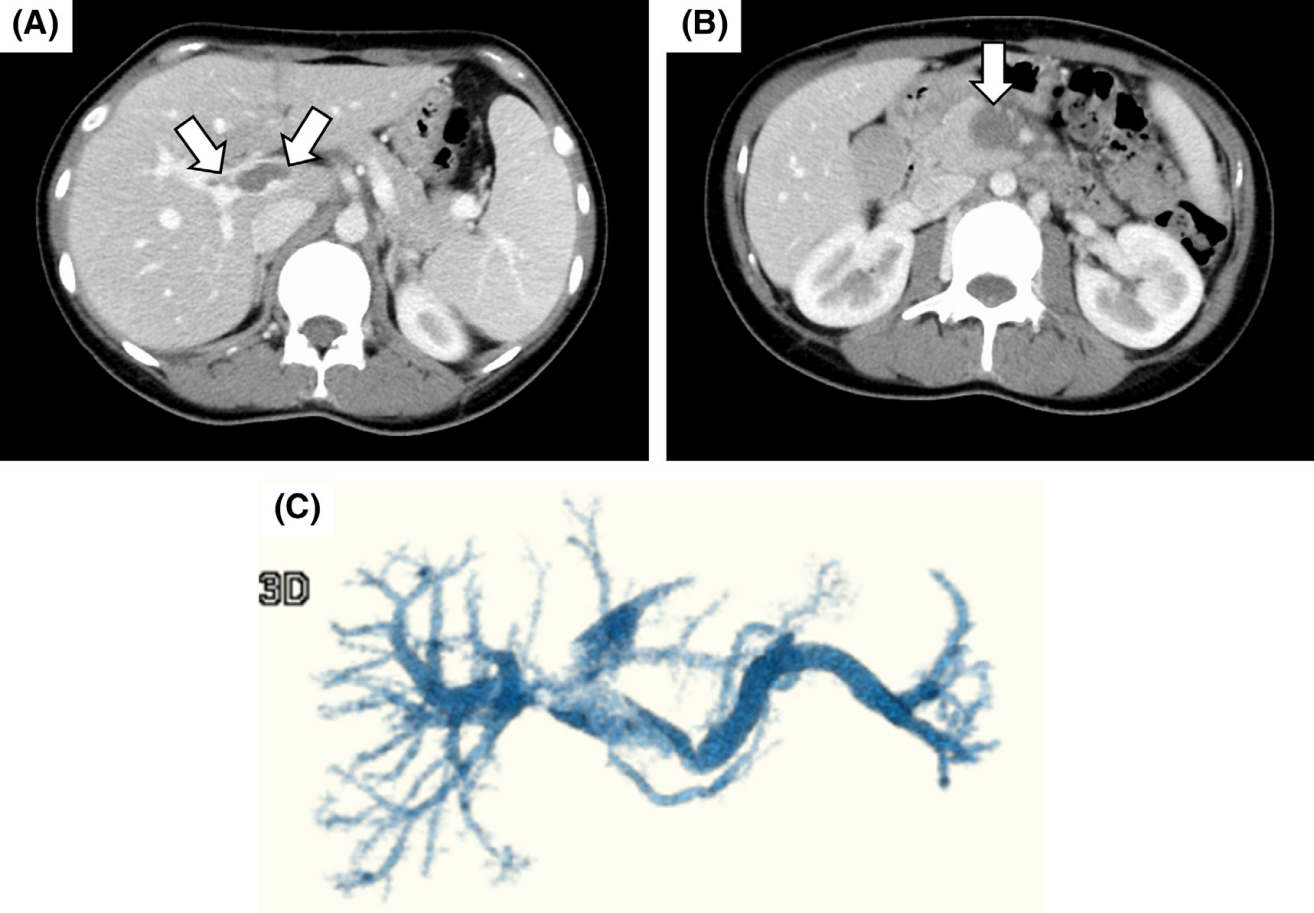
Images of contrast‐enhanced computed tomography (CT) on the day of admission. (A) A massive intrahepatic thrombus (arrows) is observed in addition to splenomegaly. (B) The thrombus occludes the superior mesenteric vein (SMV) (arrow), and no contrast flow is observed. (C) A three‐dimensional reconstructed image of the portal venous system obtained using contrast‐enhanced CT images failed to depict SMV, with partial loss of depiction of the intrahepatic portal vein.

**Figure 3:**
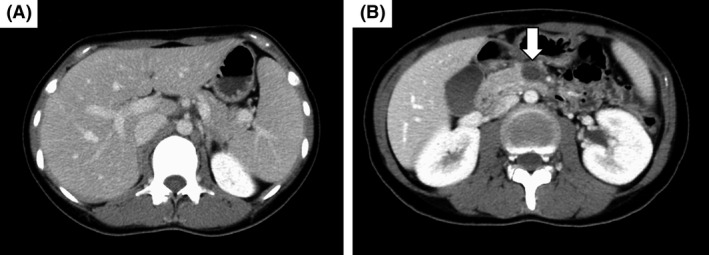
Images of contrast‐enhanced computed tomography (CT) on day 7. (A) Complete resolution of the intrahepatic thrombus is seen. (B) The thrombus in the superior mesenteric vein persisted (arrow), but decreased in size compared with images on the day of admission.

**Figure 4:**
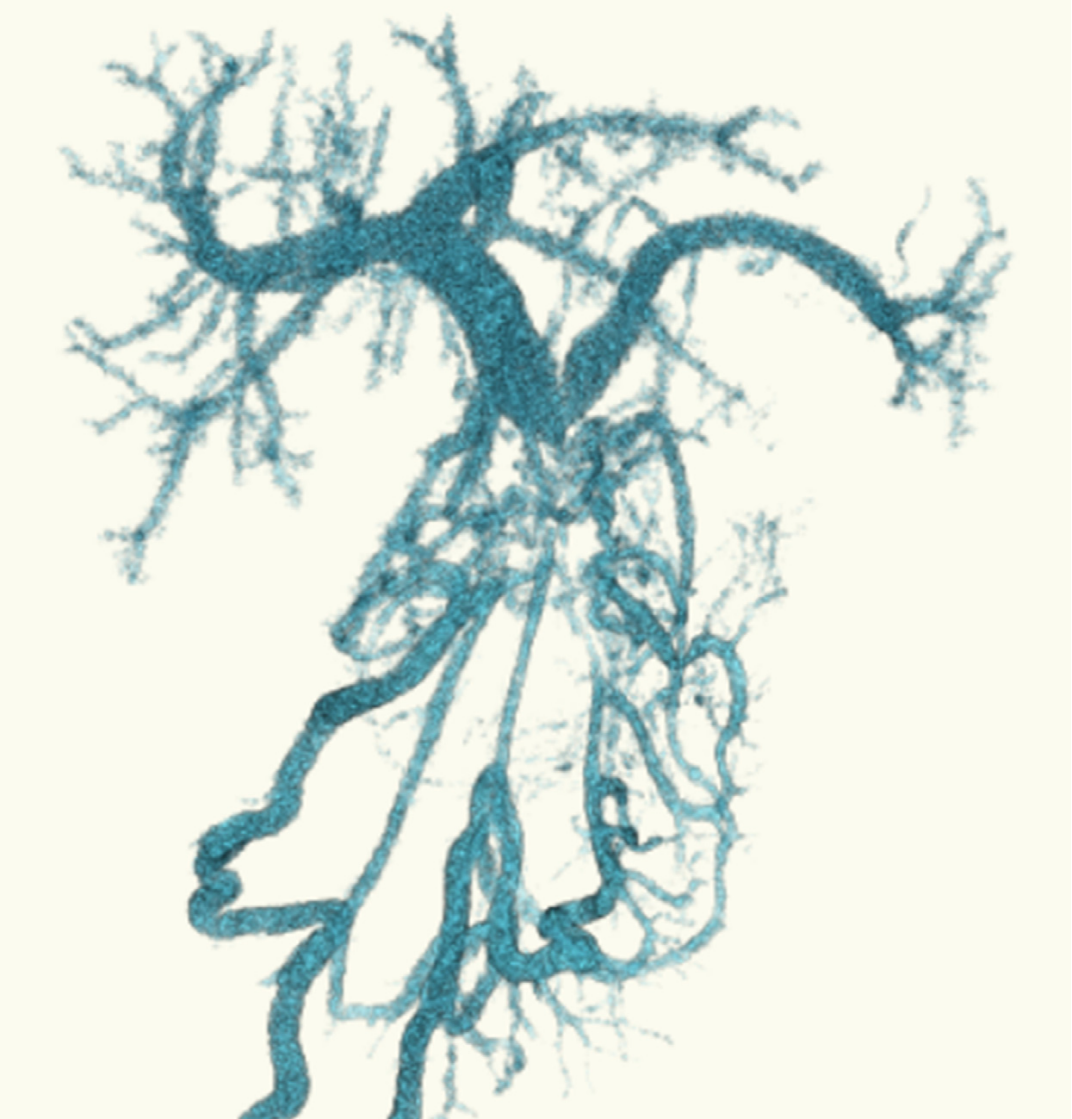
A three‐dimensional reconstructed image of the portal venous system obtained using contrast‐enhanced computed tomography images at 8 months after discharge. The superior mesenteric vein is occluded, and multiple venous collateral circulations are observed.

A 29‐year‐old primigravida Japanese woman with no remarkable past medical history was admitted at 25 weeks of gestation due to preterm premature rupture of membranes (pPROM). Bed rest, antibiotic therapy, and tocolysis with intravenous ritodrine and magnesium sulfate administrations were performed. The patient was hospitalized until delivery and was placed on bed rest. At 36 weeks of gestation, emergent CS was performed because inhibiting uterine contractions became difficult despite the administration of tocolytic agents and because the fetus was in the breech position. The surgery was performed without complications, and a healthy neonate was delivered (female baby, 2704 g, with Apgar score of 10 at 1 and 5 min). During surgery, amniotic fluid was clear without any signs of chorioamnionitis. Because the patient was not at a high risk of venous thromboembolism, anticoagulation was not performed during hospitalization and during the perioperative period. The patient remained afebrile, and no sign of infection was observed during the perioperative period. The patient developed an acute onset of epigastric pain 5 days after CS. The pain was temporally relieved by oral administration of H2‐blocker; the patient was discharged on the same day. However, the pain continued after discharge and was exacerbated by dietary intake. On a follow‐up visit 1 week after discharge, she was distressed due to the pain. At presentation, her body weight was 52.0 kg, height was 160.0 cm, and body mass index was 20. 3 kg/m^2^. Her vital signs included body temperature of 37.3°C, blood pressure of 114/69 mmHg, heart rate of 66 beats/min, respiratory rate of 16 breathes/min, and oxygen saturation of 99% with room air. On physical examination, her abdomen was not distended, although abdominal tenderness was observed. There were no indications of jaundice or edema. Blood tests revealed a white blood cell count of 12,670/*μ*L (normal range, 4000–9000/*μ*L), red blood cell count of 448 × 10^4^/*μ*L (376–500 × 10^4^/*μ*L), hemoglobin level of 11.8 g/dL (11.3–15.2 g/dL), platelet count of 37.3 × 10^4^/*μ*L (15–35 × 10^4^/*μ*L), C‐reactive protein level of 5.09 mg/dL (<0.30 mg/dL), and liver function test results within normal ranges. A coagulation test revealed partial prothrombin time‐internationalized ratio (PT‐INR) of 0.98 (0.85–1.15) and activated partial thromboplastin time (aPTT) of 32.2 sec (26–35 sec). Protein S antigen, protein C antigen, antithrombin III, and activation of factor V levels were within normal ranges. Anticardiolipin (aCL) and aCL beta‐2‐glycoprotein I antibodies showed negative results. These test results ruled out the possibility of underlying hypercoagulative disorders. Abdominal ultrasonography revealed a hyperechoic mass in the intrahepatic PV and superior mesenteric vein (SMV) (Fig. 1). Contrast‐enhanced computed tomography (CT) revealed a massive thrombus extending from SMV to intrahepatic PV and splenomegaly (Fig. 2). She was diagnosed with acute PVT, and anticoagulation therapy with intravenous unfractionated heparin (UFH) was immediately initiated while maintaining aPTT at 1.5–2.5 times the normal range. Considering intestinal ischemia due to dietary intake, fasting therapy and parenteral nutrition were also initiated. After admission, the patient's pain was gradually relieved and finally disappeared on day 3. Follow‐up contrast‐enhanced CT on day 7 showed resolution of the thrombus in the intrahepatic PV and a decrease in the size of the thrombus in SMV (Fig. 3). Dietary ingestion was initiated on the same day. Intravenous UFH was switched to oral warfarin on day 14, and PT‐INR was maintained between 2.0 and 3.0. The patient was discharged on day 30, and warfarin was continued for approximately 8 months because of the residual thrombus in SMV. Follow‐up contrast‐enhanced CT at 8 months after discharge showed the development of collaterals due to SMV occlusion (Fig. 4). Although the risk of recurrent PVT and exacerbation of symptoms were high, the patient wanted to bear a second child and became pregnant at 13 months after discharge. Subcutaneous low‐molecular‐weight heparin (LMWH) injection (5000 U/12 h) was initiated after the detection of pregnancy at 6 weeks of gestation and continued during pregnancy. PVT was followed‐up using monthly ultrasound evaluations during pregnancy, and no new PVT was observed. Later, the patient was hospitalized because of pPROM at 36 weeks of gestation. Emergent CS was performed, and successful delivery was achieved (female baby, 2611 g, with Apgar score of 9 at 1 and 5 min). LMWH was reinitiated 6 h after the surgery and continued to be used. The patient was discharged at 6 days after CS. Because the patient's previous history of PVT and repeated CS was considered to be risk factors for recurrent PVT, anticoagulation therapy was continued for 6 weeks after CS. No new PVT was observed on ultrasound evaluations 1 year after the discharge.

## Discussion

Pregnancy complicated with noncirrhotic PVT is usually associated with an underlying disease that causes a hypercoagulable state, such as protein C deficiency, protein S deficiency, antiphospholipid syndrome, factor V Leiden mutation, and myeloproliferative disorders 11; pregnancy without such complications is relatively rare. However, pregnancy itself is associated with a hypercoagulable state 4 and increased portal venous pressure 5. Although the exact mechanism remains unclear, hypercoagulability during pregnancy may be associated with hormonal changes, including increased serum estradiol level 12. During pregnancy, levels of coagulation factors, including factors VII, VIII, X, and XII, increase in addition to von Willebrand factor and ristocetin cofactor 13, 14. Decreased protein S levels also contribute to hypercoagulability, whereas protein C levels remain the same or slightly elevated during pregnancy 14, 15. Fibrinolysis is reduced during pregnancy by decreased levels of tissue plasminogen activator activity and increased levels of plasminogen activator inhibitor‐1 (PAI‐1) and PAI‐2 14, 15. Coagulability reaches peak at the time of delivery with placental expulsion due to the release of thromboplastic substances, which is a mechanism for preventing maternal blood loss 13. Moreover, increased blood flow in PV due to hemodynamic and physiological changes, which peak at 32 weeks of gestation, contributes to portal venous stasis 5. In the present case, although pregnancy and delivery were risk factors for hypercoagulability, we assumed that the thrombus was gradually formed during prolonged bed rest due to venous compression caused by the enlarged uterus and that the surgical stress of CS may have accelerated thrombus formation. Complete occlusion may have corresponded to the acute onset of epigastric pain in this patient. Diagnosing acute PVT is challenging because it sometimes manifests as acute abdomen, but color Doppler ultrasonography is a noninvasive and useful modality for the diagnosis of PVT with sensitivity and specificity ranging from 66% to 100% 16. In the present case, ultrasound evaluation at the onset of the initial symptom of epigastric pain may have likely resulted in an early diagnosis.

Although there is no consensus on the treatment for PVT, anticoagulation therapy is a reasonable and beneficial treatment option, particularly in patients with acute PVT. However, only a limited number of studies have investigated the efficacy of anticoagulation therapy for acute PVT 17, 18, 19, 20. Condat et al. 17 compared their retrospective cohort study with three previous studies and reported that anticoagulation therapy for 6 months with immediate initiation after diagnosis was associated with complete recanalization in approximately 50% of patients, partial recanalization in approximately 40%, and no recanalization in approximately 10%.

Early initiation of anticoagulation treatment is an important factor for recanalization. Turnes et al. 18 reported that 63% of patients who were administered heparin therapy within the first week of diagnosis obtained recanalization, whereas only 18% who were administered anticoagulation therapy at later stages obtained recanalization. Considering delayed recanalization, anticoagulant administration should be continued for 3–6 months if the patient has no indication of thrombotic tendencies 21. Although the benefits of anticoagulation therapy for chronic PVT remain controversial, this strategy may be effective to prevent the recurrence of thrombotic events with close monitoring of bleeding complications 19. Thrombolytic therapy was reported to be possibly effective with complete recanalization in 41% of patients and partial recanalization in 45% according to a systematic review conducted by Hall et al. 20. However, considering the high incidence of major complications (60%) 22, thrombolytic therapy should be considered only if anticoagulation therapy fails. Subcutaneous LMWH injection can be safely administered during pregnancy in patients with chronic PVT because it has the advantage of few side effects and requires no monitoring 23.

In the present case, as a consequence of chronic PVT, venous collaterals were formed to bypass the occlusion. Such cavernous transformation of PV is known as portal cavernoma 10. In the acute phase of PVT, arterial vasodilation works as a compensation for decreased PV blood flow due to obstruction 2, and portal cavernoma formation begins within a few days after PV occlusion and is completed within 3–5 weeks 24, 25. The clinical manifestations of portal hypertension include hematemesis due to esophageal varices and ascites and pancytopenia due to splenomegaly. In the current case, although the development of multiple collaterals corresponding to SMV regions was observed, there was no indication of esophageal and gastric varices on CT images, which may be explained by the decrease in PV pressure due to partial PVT resolution with anticoagulation therapy.

There have been several studies on PVT and extrahepatic PV obstruction during pregnancy 26, 27, 28, 29 [extrahepatic portal venous obstruction (EHPVO), which refers to the obstruction and cavernous formation of PV with or without the involvement of the intrahepatic PV branches; splenic; and/or SMV] 30. However, there have been no large‐scale studies on pregnancy‐associated PVT in healthy individuals. Mandal et al. 26 studied 41 pregnancies in 24 patients with EHPVO and reported that variceal bleeding during pregnancy was associated with unfavorable outcomes, such as preterm labor, low birth weight, and perinatal death. They also reported that the incidences of variceal bleedings and preterm labor were much higher in patients diagnosed with EHPVO prior to pregnancy than in those without this diagnosis (relative risk, 16.5 vs. 20.6, respectively) 26. In a retrospective multicenter study of 45 pregnancies in 25 patients with PVT treated with anticoagulation therapy on an individual basis, Hoeksta et al. 27 reported favorable fetal and maternal outcomes for 80% of pregnancies reaching 20 weeks of gestation. However, they also reported an increased miscarriage rate of 20%. Considering the risk of fatal hypoxia during these procedures, upper GI endoscopy during pregnancy is not recommended, with the exception of cases with significant variceal bleeding 31. Endoscopic therapy prior to pregnancy in patients with upper varices may lead to good clinical outcomes 5, 28. Although CT is a good screening modality to evaluate esophageal varices 32, in selective cases, such as the present case, upper GI endoscopy prior to pregnancy should be considered for the early detection of varices.

Although pregnancy and delivery contribute to hypercoagulability, PVT is a rare complication during pregnancy and postpartum period in healthy individuals. Patients who are advised prolonged bed rest for the treatment of threatened preterm labor and surgical stress due to CS might be risks for PVT. Ultrasound examination is useful for correct and prompt diagnosis and early treatment initiation to prevent the progression of portal hypertension and reduce the risk of variceal bleeding. In the present case, the thrombus in SMV was not completely resolved, and splenomegaly was observed, but the patient achieved a favorable clinical outcome with anticoagulation therapy during both the acute and chronic phases. During clinical course, no bleeding episode was observed with close monitoring. As variceal bleedings is one of the most serious complications in patients with PVT, upper GI endoscopy for the evaluation of varices might be needed in selected cases.

## Conflict of Interest

The authors declare no conflict of interest associated with this manuscript.

## Authorship

AH drafted the manuscript. KM contributed to the critical revision of the manuscript. All authors read and approved the final version of the manuscript.
